# One-Year Survival and Prognosticators of Adults With Acute Leukemia at the Uganda Cancer Institute

**DOI:** 10.1200/GO.22.00244

**Published:** 2023-07-27

**Authors:** Barbra Natukunda, Abrahams Omoding, Felix Bongomin, Kelvin Roland Mubiru, Henry Ddungu, Christine Sekaggya-Wiltshire, Harriet Mayanja-Kizza

**Affiliations:** ^1^ Uganda Cancer Institute, Kampala, Uganda; ^2^ Department of Medicine, School of Medicine, College of Health Sciences, Makerere University, Kampala, Uganda; ^3^ Department of Medical Microbiology and Immunology, Faculty of Medicine, Gulu University, Gulu, Uganda; ^4^ Hutchinson Centre Research Institute—Uganda, Kampala, Uganda

## Abstract

**PURPOSE:**

Acute leukemias are associated with substantial morbidity and mortality, particularly in the adult population. Despite an increasing burden of acute leukemia in developing countries, there are limited data on clinical outcomes and prognostic factors in this setting. In this study, we aimed to describe the clinical characteristics, survival, and prognostic factors of adults with acute leukemia at the Uganda Cancer Institute (UCI).

**METHODS:**

A retrospective cohort study was conducted between January 2009 and December 2018, reviewing data of patients 18 years or older with a cytopathologic diagnosis of acute leukemia at UCI. Data were extracted on clinical and laboratory characteristics, response to treatment, and survival. Cox-proportional hazards regression and survival analysis were performed to determine survival rates and associated factors. *P* < .05 was considered statistically significant.

**RESULTS:**

In total, 233 participants were enrolled. Most (59.2%. n = 138) participants were male, with a median age of 32 years (IQR, 23-48 years), and 136 (58.4%) had AML. Overall, the 1-year survival was 16.5%, with a median survival time of 47 (IQR, 21-219) days. Predictors of mortality were being a female (adjusted hazard ratio [aHR], 2.8; 95% CI, 1.2 to 6.7; *P* = .022) and overweight (aHR, 4.2; 95% CI, 1.3 to 13.4; *P* = .015). Among the patients who had AML, the predictors were poor Eastern Cooperative Oncology Group (ECOG; aHR, 3.1; 95% CI, 1.6 to 6.2; *P* = .001) and HIV (aHR, 6.0; 95% CI, 1.7 to 20.5; *P* = .004). Among the patients who had ALL, the predictors were poor ECOG (aHR, 2.3; 95% CI, 1.3 to 4.1; *P* = .006).

**CONCLUSION:**

Patients with acute leukemia in Uganda have poor overall survival. Prospective studies are recommended to better understand causes of early mortality.

## Teaser

In a retrospective review of 233 adults with acute leukemia (136 [58.4%] AML) treated at the Uganda Cancer Institute (UCI) between January 2009 and December 2018, the overall survival at one month was 64.8%, at 6 months, 30.0%, and at one year, 16.4%.

## BACKGROUND

Acute leukemias pose a substantial clinical burden worldwide, with rising incidence but poor overall survival rates, particularly with increasing age.^1^ In sub-Saharan Africa, an increasing incidence of leukemia among young adult age groups has been reported to affect manpower and economic growth.^2^ In Uganda, there has been a 33% increase in the incidence of leukemia between 1991 and 2010.^3^ Furthermore, it is predicted that the incidence of acute leukemia in adults may continue to rise because of occupational and environmental exposures to pollutants, cigarette smoking, and the prescription of alkylating agents to young people with malignant disease.^4^

**CONTEXT**

**Key Objective**
What are the clinical characteristics, survival, and prognostic factors of adults with acute leukemia at the Uganda Cancer Institute, Uganda?
**Knowledge Generated**
This study showed that only about one in six patients with acute leukemias in Uganda live for 12 months, with more than half dying with the first 50 days of diagnosis. Female sex and those with overweight, poor functional status, and HIV infection were associated with poor prognosis.
**Relevance**
We provide baseline data to inform further studies and interventions on early diagnosis, better diagnostics to confirm and risk stratify acute leukemia, and standardization of therapeutics and treatment response evaluations.

Survival of adults with acute leukemia has remarkably improved in high-income settings over the past few years because of better diagnostics, advances in treatment protocols, utilization of stem cell transplantation, excellent supportive care, and well-established government-funded public health care systems. On the one hand, AML is cured in 35%-40% of adult patients who are 60 years or younger and in 5%-15% of patients who are older than 60 years.^5^ On the other hand, cure rates for adults with ALL in resource-rich settings (RRS) also approach 40%.^6^

However, in resource-limited settings (RLS), patients often present with extensive disease, which is, at least partly, a consequence of delay in diagnosis,^7^ and there is poor access to acute and supportive care during treatment.^8^ The overarching aim of this study was therefore to describe the characteristics, survival, and prognostic factors of adult patients with acute leukemia at a regional center of excellence in oncology situated in Uganda.

## METHODS

### Study Design

This was a descriptive, retrospective, chart review study. All available charts of adult patients admitted with a diagnosis of acute leukemia from January 2009 to December 2018 at the Uganda Cancer Institute (UCI) were reviewed. Acute leukemia was defined as a diagnosis of either AML or ALL after morphological demonstration of the presence of >20% myeloblast or lymphoblast cells on bone marrow aspirate for AML and ALL, respectively.

### Study Setting

UCI is the national cancer referral center and manages a majority of patients with cancer in Uganda and some patients from neighboring countries. Patients with hematologic malignancies are admitted to the lymphoma treatment center (LTC) ward, which has a bed capacity of 25 beds. On average, three new adult patients with acute leukemia are received every month on the LTC ward; on receipt, confirmatory tests are performed, if necessary; and thereafter, other laboratory and radiologic investigations are performed before treatment initiation.

### Study Population

We included participants age 18 years or older, who had a confirmed diagnosis of an acute leukemia on the basis of peripheral blood film or bone marrow aspirate and were registered at the UCI during the study period. Patients who received any acute leukemia chemotherapy before admission to UCI or who had the blast phase of chronic leukemia were excluded.

### Study Variables

For each chart, we extracted data on vital status (dead, alive, or lost to follow-up) and duration between acute leukemia diagnosis and death (for those who are died) or lost to follow-up.

### Sample Size Estimation

Using the Kish and Leslie (1965) formula for a descriptive objective in a single population proportion and adjusting for 10% missing data on the outcome variable, a minimum of 195 participants were needed to answer the objective on characteristics of adult patients with acute leukemia admitted to UCI. We used the sample size formula by Schoenfeld (1983) for the proportional hazards regression model and calculated a minimum of 168 participants required to answer the objective on the survival and prognostic factors of adult patients with acute leukemia admitted to UCI.

### Data Collection

The patient register at the UCI Records office was used to identify all patients with a diagnosis of acute leukemia who were 18 years or older during the study period. Data were extracted using a pretested data abstraction form for the following: patient demographics, clinical symptoms at presentation, physical examination findings at intake, medical and social history, diagnostic tests, hospitalization and induction treatment, treatment response, and overall outcomes. Patients whose vital status could not be obtained from UCI records (patient chart or death register book) were contacted directly or through their next of kin via phone calls. Patients whose vital status could not be obtained from UCI records and could not be reached on phone were be defined as lost to follow-up.

### Data Analysis

Data were analyzed using STATA Version 16.0. Descriptive statistics such as proportions and frequency distribution were used to summarize categorical variables. Continuous variables were presented in terms of medians and IQR. Kaplan-Meier methods were used to estimate overall survival with 95% CIs at 1 year. Survival time was defined as the time from diagnosis of acute leukemia to death.

All factors associated with survival were analyzed using bivariate analysis (cox regression) and multivariable analysis (Cox-proportional hazards regression model). Variables that had *P* < .2 from bivariate analysis were included in the multivariable analysis. The multivariable cox regression model was tested for proportionality using the Schoenfeld residuals. For models that violated the proportionality assumption, we used stratified cox model analysis. The threshold for statistical significance was set to a two-tailed α < .05.

Sensitivity: Sensitivity analysis was used for those who were lost to follow-up in which case they were categorized as dead a month after their last recorded visit.

This study was conducted in accordance with the ethical principles stated in the *Declaration of Helsinki*. The Makerere University School of Medicine and Ethics Committee approved the study protocol and provided a waiver of consent (approval number #REC REF 2020-157).

## RESULTS

**FIG 1:**
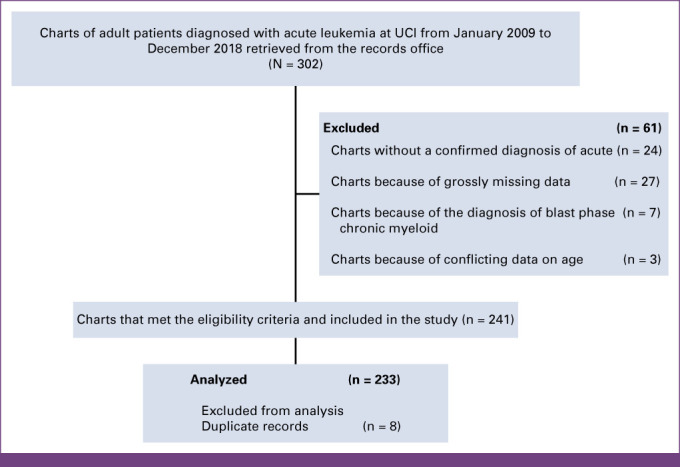
Flowchart. UCI, Uganda Cancer Institute.

A total of 302 charts were retrieved. The charts were screened, and 61 were excluded (Fig 1).

### Patient Characteristics

A total of 233 records of adult patients with a confirmed diagnosis of acute leukemia were included in this study. Table 1 summarizes the demographic characteristics of the patients. The median age was 32 years (IQR, 23-48). Participants with AML were older than those with ALL (median age, 38 years *v* 26 years). A majority (59.2%, n = 138) of patients were male, and 45.5% (n = 106) were in an informal employment.

**TABLE 1:**
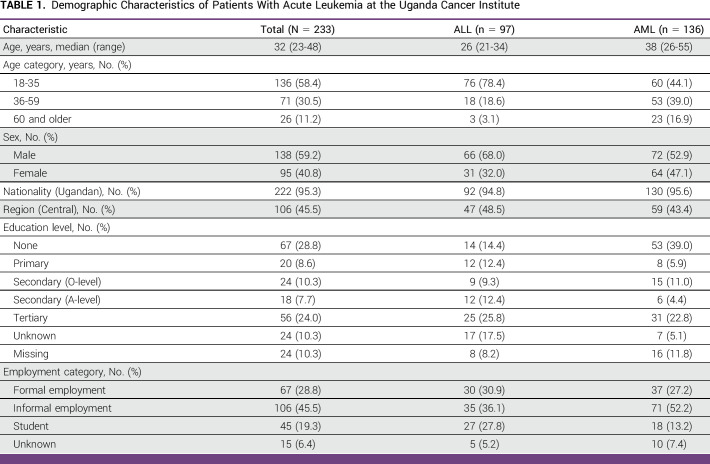
Demographic Characteristics of Patients With Acute Leukemia at the Uganda Cancer Institute

### Acute Leukemia Diagnosis and Classification

The most frequent diagnosis was AML (n = 136, 58.4%) followed by ALL (n = 97, 41.6%).

#### Classification

**FIG 2:**
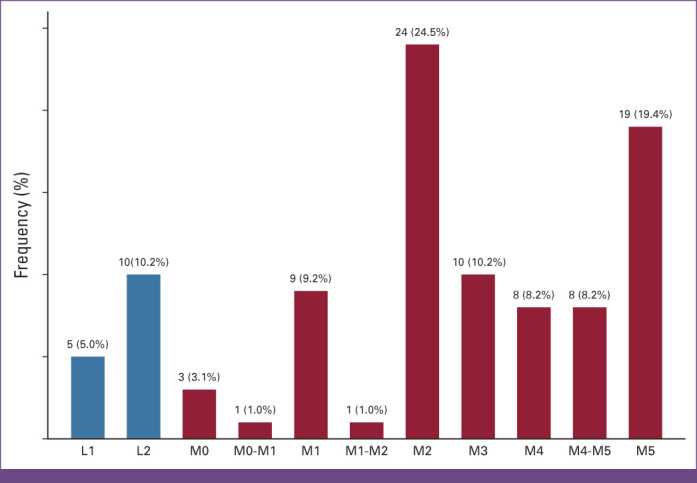
FAB classification of patients diagnosed with acute leukemia at the Uganda Cancer Institute (n = 98). FAB, French American British; L, acute lymphoblastic leukemia; M, acute myeloid leukemia.

Of the 233 study participants, 42% (n = 98) had a French American British (FAB) classification documented. 24.5% (n = 25) and 19.4% (n = 19) with AML had M2 and M5, respectively (Fig 2). Seventeen (7%) patients had a Philadelphia chromosome test performed at admission into UCI, and this test was positive in 11 of these patients (64.7%). Seven patients (2.9%) had flow cytometry test results. Only 0.8% (n = 2) had cytogenetic testing; one had a normal karyotype AML and FLT3 by internal tandem duplication. The second had PML/RARA fusion gene, and 80% of cells were positive for translocation *t*(15;17) (q24;q21).

### Clinical Presentation

The most common presenting complaints were fever (77.4%), body weakness (73.5%), palpitation (59.6%), bleeding (53.0%), and weight loss (45.9%). One hundred and twenty-two patients with acute leukemia presented with abnormal bleeding, a majority (57.4%) of these patients had AML, and bleeding sites included epistaxis 40% (n = 50), the buccal cavity 38% (n = 47), under the skin, per vagina, conjunctiva, stools, and ears. 1.3% (n = 3) of patients presented with convulsions, and they all had a diagnosis of ALL. A minority of patients (≤2%) presented with other complaints such as chest pain, lower limb swelling, painful testicular swelling, dizziness, and loose stools. The Data Supplement summarizes the main presenting complaints of patients with acute leukemia at UCI. The Data Supplement summarizes family history of cancer and comorbidities of participants diagnosed with acute leukemia at UCI.

### Physical Examination, Laboratory, and Imaging Findings

On physical examination, it is found that a majority of patients (59.5%, n = 100) had a normal BMI and 20.2% of the patients were underweight. A total of 168 (72.1%) participants had an Eastern Cooperative Oncology Group (ECOG) score of ≥2. Only 24.0% (n = 56) of the patients were asymptomatic or symptomatic but fully ambulatory at the time of admission into UCI (Data Supplement).

The median total white blood cell count was 28.2 × 10^9^ cells/mm^3^. Total WBC count was significantly elevated among patients with ALL compared with those diagnosed with AML (*P* = .024). A majority of patients had anemia (95.2%, n = 222) with a median hemoglobin level of 6 g/dL (IQR, 4.8-7.7), and 91.8% (n = 214) of patients had thrombocytopenia. Overall, 17.2% of patients with acute leukemia had pancytopenia, and of these, 60% had a diagnosis of AML. Of the patients, 58.3% (n = 136) had elevated lactate dehydrogenase (LDH) with a median LDH level of 550.6 U/L (normal range LDH, 125-220 IU/L).

On ultrasonography, 57.0% (n = 133) of patients had hepatomegaly, 61.8% (n = 114) had splenomegaly, and 12.4% (n = 29) had ascites. And 10.7% (n = 25) of patients had evidence of pleural effusion on chest x-ray. The above features, suggestive of extramedullary disease, were significantly higher among patients with ALL than those with AML (*P* = .001).

### Management of Adult Patients With Acute Leukemia at UCI

The median duration of hospitalization at UCI was 20 days (IQR, 10-33.5 days). Of the patients, 72.5% received intravenous and/or oral chemotherapy, whereas 12.8% (n = 30) received intrathecal chemotherapy. A majority of patients (88.4%, n = 206) received antibiotics; metronidazole (n = 113) and ceftriaxone (n = 78) were the most used antibiotics. Of the patients, 84.9% (n = 198) received blood transfusion, and 50.6% (n = 118) received platelet transfusion. Palliative care consultation was documented in only 3.8% (n = 9) of the patient charts during first admission to UCI (Data Supplement).

Chemotherapy receipt: The average number of days from diagnosis to treatment among patients was 19 (IQR, 7-13.5) and 48 (IQR, 8-18) for ALL and AML, respectively. For induction treatment, a majority of patients (63.2%, n = 86) with AML received daunorubicin- and cytarabine-based regimens, whereas 65% (n = 63) of the patients with ALL received linker's regimen with daunorubicin, vincristine, L-asparaginase, and prednisolone. Sixty-four patients (27.4%) did not receive chemotherapy. Fewer patients with ALL (39%, n = 30 *v* 79.8%, n = 71 [AML]) completed their induction chemotherapy as shown in the Data Supplement. Treatment response after completion of first induction chemotherapy was not known in more than a third of all patients, Data Supplement.

### Survival of Patients With Acute Leukemia

**FIG 3:**
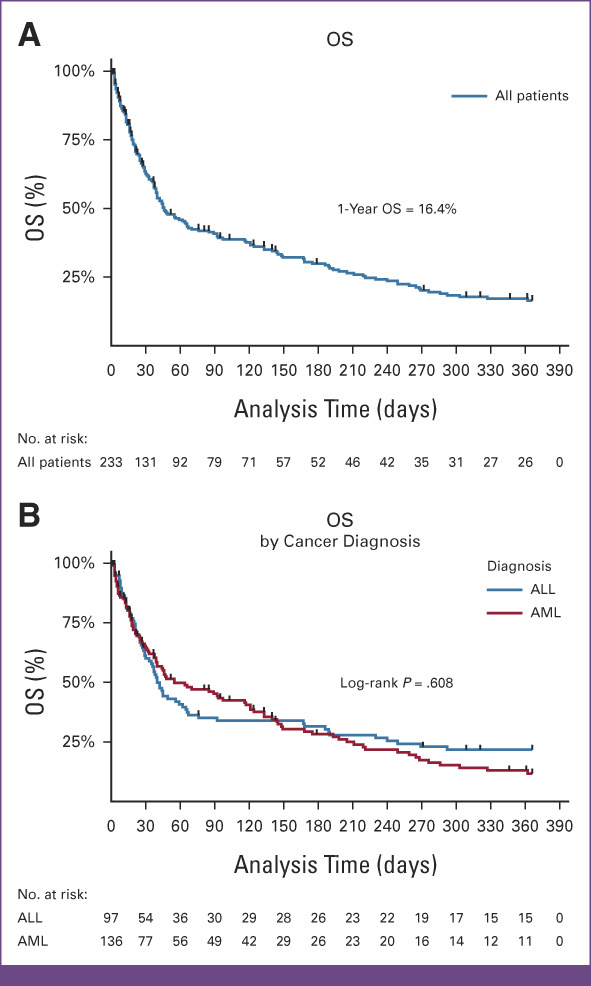
(A) OS of adult patients with acute leukemia at the UCI. (B) Survival of adult patients with acute leukemia at the UCI. OS, overall survival; UCI, Uganda Cancer Institute.

**FIG 4:**
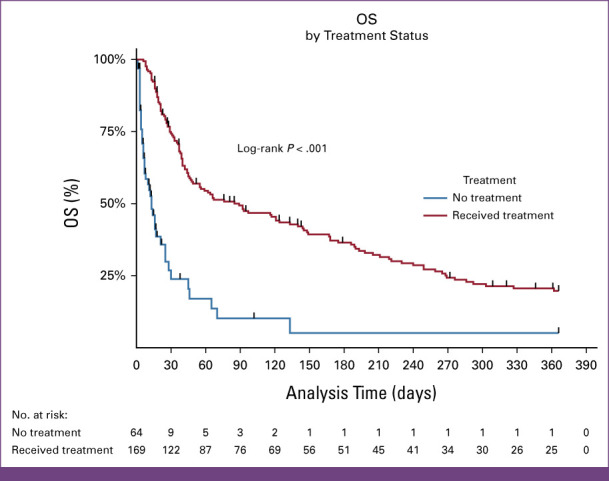
Chemotherapy treatment status: OS of adult patients with acute leukemia at the Uganda Cancer Institute. OS, overall survival.

Overall, the median survival time after diagnosis with acute leukemia was 46 days (IQR, 18-221 days). The overall survival at 1 month was 64.8% (95% CI, 58.0 to 70.7), at 6 months, it was 30.0% (95% CI, 23.8 to 36.5), and at 1 year, it was 16.4% (95% CI, 11.5 to 22.3; Fig 3A). The median survival time for patients with AML was 55 days (IQR, 18-205 days), whereas it was 40 days (IQR, 20-249 days) for patients with ALL. The difference in survival time between patients with AML and ALL was not statistically significant in the log-rank test, *P* = .608 (Fig 3B). Patients who received any chemotherapy treatment survived better than those who received no chemotherapy; the median overall survival was 89 days versus 13 days, respectively, as shown in Figure 4.

### Prognostic Factors After Diagnosis of Acute Leukemia

In multivariable analysis, being female (hazard ratio [HR], 2.8; 95% CI, 1.2 to 6.7; *P* = .022), overweight (HR, 4.2; 95% CI, 1.3 to 13.4; *P* = .001), and a refractory response after first induction (HR, 2.8; 95% CI, 1.0 to 8.2; *P* = .063) were significantly associated with poor survival (Table 2). Patients with high ECOG score (3,4) were 1.4 times more likely to die from acute leukemia compared with those with low ECOG score (HR, 1.4; 95% CI, 0.4 to 4.7; *P* = .561).

**TABLE 2:**
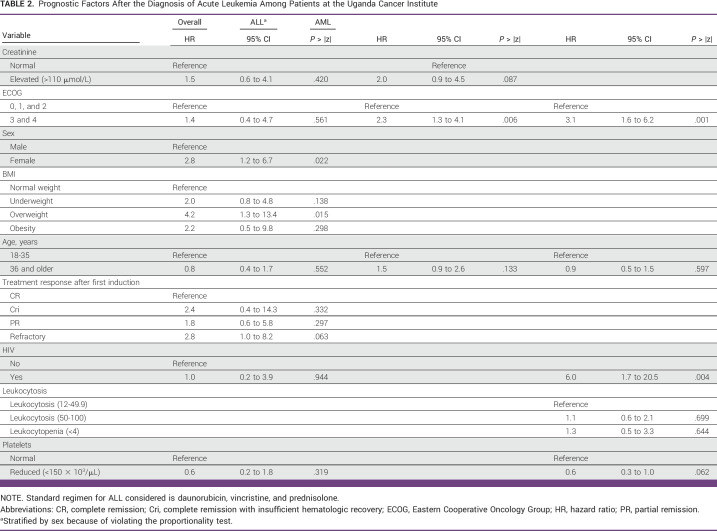
Prognostic Factors After the Diagnosis of Acute Leukemia Among Patients at the Uganda Cancer Institute

## DISCUSSION

In this retrospective study at UCI, most patients had a diagnosis of AML (58.4%) and were male (59.2%). Survival after diagnosis with acute leukemia was poor; the 1-year overall survival was 16.4%. Factors significantly associated with improved survival included being male, HIV-negative status, normal BMI, low ECOG status, and receipt of chemotherapy with complete response.

In the present study, the most prevalent diagnosis was AML (56.3%), which is consistent with other studies that show that AML is the most common acute leukemia in adults.^9^ The median age in our study was 32 years unlike the worldwide median age of 68 years at diagnosis of adult acute leukemia; up to 58.4% of our patients were age 18-35 years, which is also consistent with other studies in RLS that show that median age of adults with acute leukemia is lower than those in RRS.^10-12^ Younger age at diagnosis could be explained in part because of the lower average life expectancy in our setting and probable selection bias where younger patients are referred to the larger national hospitals. Patients older than 60 years have been reported to have worse outcomes than younger patients regardless of cytogenetics or prognostic classification.^13^ However, in our study, younger age only conferred a better prognosis in univariate but not in multivariable analysis. This is probably because of an epidemiologic difference in the population of patients in our setting. Sixty percent of the patients in this study were male, which is similar to other studies that have reported male predominance of acute leukemia.^14-16^ Interestingly, females were more likely to die from acute leukemia, which is unlike previous studies that suggest that in adults, males have worse survival from both acute leukemia^17^ and other cancers,^18^ the reasons for which finding are yet to be investigated.

In this study, a majority of patients presented after at least 1 month of symptoms and had features suggestive of extramedullary disease. In RRS, with universal health care systems, low education level^19^ and socioeconomic status^20^ have been shown to contribute to affect survival in younger patients with AML. In our study, only 24% and 28.8% of patients had tertiary education and formal employment, respectively. The impact of the education level on outcomes of patients with acute leukemias in RLS like Uganda where there is no universal health care system remains an area for further studies. However, limited skilled personnel, delayed and inaccurate diagnoses, often grossly inadequate histopathologic services, and infrastructure are common challenges faced in RLS, and these contribute to disparities in cancer outcomes.^21-24^ Over two thirds (68.6%, n = 157) of the patients in this study had an elevated WBC with a median value of 28.2 × 10^9^ cells/mm^3^, whereas 17% of the patients had pancytopenia. Although not common, studies have shown that patients with AML may present with pancytopenia.^25^ In ALL, pancytopenia has been reported as an independent predictor of better survival given significantly lower incidence of bulky disease at presentation.^26^ Abnormal cell counts in a patient with prolonged symptoms should raise suspicion for acute leukemia and prompt further workup.

In RRS, significant improvements in 1-, 5-, and 10-year survival of adults with acute leukemia, particularly for those younger than 60 years, have been reported since 1997.^27^ But survival after acute leukemia diagnosis remains poor or largely unpublished in many RLS, for example, the median survival for patients with acute leukemia in Pakistan was 11 months, and it was 11.5 months in Egypt.^28,29^ In our study, survival was dismal in comparison the above. Inadequate risk stratification may explain this finding as it affects management. Patients were classified using the FAB system rather than the WHO criteria, which has prognostic importance and incorporates cytogenetics and immunophenotype in classifying patients as to whether they have good or poor risk. Only 3.81% of patients in this study were classified as good/standard risk, and 30.51% as poor/high risk, and the remaining majority (65.68%) had no classification indicated; however, there was no standard tool recorded in the patient charts on which classification was defined. In addition, 63.2% of patients with AML received daunorubicin and cytarabine treatment, whereas among those with ALL, 65% received Linker's regimen. This suggests that patients received uniform chemotherapy regimens despite stratification and that a number of terminal patients (given that the median overall survival was 47 days) received chemotherapy, which is associated with harm rather than survival benefit.^30,31^ Disproportionate patient to infrastructure ratios in RLS^32^ often necessitate dose modification to balance intensity of cytotoxic treatment intended for cure with supportive therapies. Jain et al^33^ reported that in RLS, intensified chemotherapy regimens are associated with a significant increase in treatment-related toxicity and cost, yet alternative regimens can provide comparable clinical outcomes. Therefore, through prospective studies, it may be possible to develop local evidence-based guidelines for risk stratification and chemotherapy modifications. Nonetheless, studies in neighboring countries where low-dose intense treatments were used in patients with acute leukemia showed no higher survival. In a small study of 20 patients with AML receiving low-dose cytarabine in Kenya, only 64.7% (n = 11) of patients completed one full cycle of treatment and 75% (n = 15) died within 1 year of diagnosis.^34^ Still, in Kenya, a larger study of 113 patients with AML whose median age was 40 years and in which no patient received standard intensive induction chemotherapy because of inadequate supportive care showed that the median overall survival after diagnosis was only 45 days.^10^ Another study showed that although toxicity was acceptable with the use of a low-intensity regimen among children with ALL, more than 70% of patients had died at 2 years and disease relapse was a substantial cause of failure.^35^ Patient population heterogeneity therefore remains to be considered as a contributor to the outcome of patients with acute leukemia. The use of targeted therapies^36-38^ and participation in clinical trials have been shown to confer survival benefit to participants in RRS because of the use of stringent treatment protocols, superior observation, and supportive care.^39,40^ However, selection bias and use of superior protocols may account for this finding.^41^

Although only 4.8% of patients in our study had HIV, these patients were twice as likely to die from acute leukemia. This is in agreement with the data published by Evans et al^42^ who reported that patients with AML with HIV had poor tolerance to chemotherapy and a short disease-free survival. Our study also showed that patients with a poor ECOG score had poor survival, and it is important to note that performance status in itself is likely to reflect the severity of leukemia and has been shown to have a major impact on chance of complete remission (CR), short-term survival, and long-term survival (independent of patient comorbidities).^43^ Patients who were underweight, overweight, and obese had poorer survival than those with normal BMI, which is in agreement with other studies that have shown a trend toward poor overall survival in overweight/obese patients with leukemia.^44,45^ Comorbid management and supportive therapies for very ill patients remain an important consideration in our setting.

To our knowledge, this is the first study on characteristics, survival, and prognostic factors of acute leukemia among adults over a 10-year period in Uganda. Our findings inform prospective studies on acute leukemia in Uganda. However, this was a retrospective chart review study and a number of missing fields from patient files were found. In addition, certain important factors associated with survival could not be assessed through this retrospective chart review study, which included compliance to treatment, dose of treatment, cancer and host biology, and circumstances at death, among others.

In conclusion, at the UCI, acute leukemia overall survival is poor with less than one in five surviving for 1 year after diagnosis. Being female, overweight, having a poor ECOG score, and failure to achieve CR after first induction chemotherapy are factors associated with poor outcomes of acute leukemia. We propose prospective studies to better understand causes of early mortality and poor overall survival among adult patients with acute leukemia in Uganda. From this study, we also recommend sensitization and early diagnosis, better diagnostics to confirm and risk stratify acute leukemia, and standardization of therapeutics and treatment response evaluations.

## Data Availability

The data sets used and/or analyzed during the current study are available from the corresponding author on reasonable request.
